# Mobile Health App for Adolescent Asthma Self-Management: Development and Usability Study of the Pulmonary Education and Knowledge Mobile Asthma Action Plan

**DOI:** 10.2196/64212

**Published:** 2025-04-24

**Authors:** Xing He, Jiang Bian, Ariel Berlinski, Yi Guo, A Larry Simmons, S Alexandra Marshall, Carolyn J Greene, Rita Hudson Brown, Jessica Turner, Tamara T Perry

**Affiliations:** 1 Department of Biostatistics and Health Data Science, Indiana University, Indianapolis, IN, United States; 2 Regenstrief Institute, Indianapolis, IN, United States; 3 Department of Pediatrics, University of Arkansas for Medical Sciences, Little Rock, AR, United States; 4 Arkansas Children’s Research Institute, 13 Children's Way, Little Rock, AR, 72202, United States, 1 5014751183; 5 Department of Health Outcomes & Biomedical Informatics, University of Florida, Gainesville, FL, United States; 6 Arkansas Children’s Hospital, Little Rock, AR, United States; 7 Department of Health Behavior & Health Education, University of Arkansas for Medical Sciences, Little Rock, AR, United States; 8 United States Department of Veterans Affairs, Mann-Grandstaff VA Medical Center, Spokane, WA, United States

**Keywords:** asthma self-management, mobile health, user-centered design, usability, adolescents, mobile phone

## Abstract

**Background:**

Adolescents with asthma are vulnerable to poor asthma outcomes due to inadequate self-management skills and nonadherence to medications. Mobile health (mHealth) apps have shown promise in improving asthma control, medication adherence, and self-efficacy. However, existing mHealth asthma apps lack personalization and real-time feedback and are not tailored for at-risk adolescents.

**Objective:**

This study aimed to design, develop, and test a smartphone-based mHealth Asthma Action Plan for adolescents, called Pulmonary Education and Knowledge Mobile Asthma Action Plan (PEAK-mAAP), in preparation for a large-scale randomized controlled trial.

**Methods:**

We employed user-centered design principles to develop our app, leveraging our previous work and following guidelines from the National Heart, Lung, and Blood Institute. The app consists of a patient-facing mobile app and a provider-facing portal. A convenience sample of 13 adolescents (aged 12‐20 years) was recruited from the Arkansas Children’s Research Institute database or direct health care provider referrals. Participants underwent a task-based usability assessment followed by the System Usability Scale assessment to measure user satisfaction, interface effectiveness, and overall system usability.

**Results:**

PEAK-mAAP integrates 7 core modules supporting personalized asthma self-management, symptom monitoring, medication tracking, and real-time feedback. The mean System Usability Scale score was 83/100 (SD 5.54), indicating high user satisfaction and system usability. Notably, older adolescents (>17 years) reported higher usability scores (87.5) than younger users (77.5), suggesting potential age-related differences in app navigation and engagement.

**Conclusions:**

The results demonstrate that PEAK-mAAP is a feasible and user-friendly mHealth intervention for adolescent asthma self-management. While the high usability score reflects a positive user experience, some participants encountered initial usability challenges, highlighting the need for minor refinements and user training materials. The integration of personalized self-management tools and real-time feedback distinguishes PEAK-mAAP from existing asthma apps, addressing key barriers to adherence and engagement. Moving forward, an ongoing randomized controlled trial will assess its clinical effectiveness, long-term engagement, and impact on asthma outcomes, providing further insights into its potential as a scalable solution for adolescent asthma care.

## Introduction

According to the Global Asthma Report 2022 [1], asthma impacts 262 million individuals globally across all ages. The report highlighted that 1 in 10 children have asthma symptoms, and half have poorly controlled symptoms. Like most chronic diseases, a proactive way to mitigate the cost and adverse effects caused by asthma is to equip patients with effective self-management strategies [2]. Earlier investigations of mobile health (mHealth) asthma management apps show a positive effect on asthma control, medication adherence, and self-efficacy [3,4]. mHealth technology that combines personalized health management into patients’ daily routines can effectively enhance patients’ asthma self-management [4]. For instance, Farzandipour et al [5] designed a smartphone-based asthma self-management app with educational content, data recording, reminders, and physician communication features, which was rated as “good” in usability and positively impacted patients’ self-management knowledge. Kosse et al [6] found that an mHealth intervention significantly improved medication adherence in adolescents with asthma who had poor adherence rates, suggesting that health care providers should consider a tailored mHealth approach in their treatment. Fedele et al [7] conducted a randomized controlled trial (RCT) to test the feasibility and efficacy of a mHealth intervention to enhance caregiver support and asthma self-management in early adolescents, revealing clinically significant improvements in asthma control. Nevertheless, existing mHealth asthma apps lack personalization and real-time feedback, and are not tailored for at-risk adolescents. Furthermore, recent review highlights a significant lack of large-scale RCTs evaluating the efficacy and sustainability of eHealth and mHealth interventions in adolescents and young adults with chronic diseases, including asthma [8].

Adolescents are at high risk of adverse asthma outcomes due to multiple factors, such as (1) their self-management skills are often insufficient, which may lead to nonadherence to medication; and (2) adolescence is typically when there is a transition from dependence on parental supervision to medical autonomy [9]. Current practices should ensure adolescents have the correct knowledge, beliefs, and positive attitudes to self-manage their illness [10]. National Heart, Lung, and Blood Institute (NHLBI) recommends using Asthma Action Plans (AAPs) to facilitate asthma self-management [11]. In our previous studies [12,13], we developed a smartphone-based mobile health Asthma Action Plan (mAAP) tailored for adolescents. Our findings demonstrated its feasibility as a self-management tool, significantly benefiting adolescents with uncontrolled asthma. Leveraging the insights from these feasibility studies, we adopted a user-centered design (UCD) [14] approach, collaborating with a diverse group of stakeholders (ie, adolescents with asthma, their parents, clinicians, nurses, and researchers) to iteratively redesign and re-engineer the mAAP and named it, Pulmonary Education and Knowledge Mobile Asthma Action Plan (PEAK-mAAP). Critically, we designed PEAK-mAAP to address specific barriers to adopting health behaviors based on the well-established health belief model (HBM) [15]. Built on the Apache Cordova (Adobe Systems) [16] mobile app framework, PEAK-mAAP has cross-platform compatibility to meet the needs of Android (Google), iOS (Apple Inc), and web users. Complementing PEAK-mAAP, we introduced a web portal for health providers and guardians, allowing them to oversee the adolescents’ engagement with PEAK-mAAP. In this paper, we detail the design, development, and usability assessment of PEAK-mAAP.

## Methods

### Design of PEAK-mAAP

**Figure 1.**
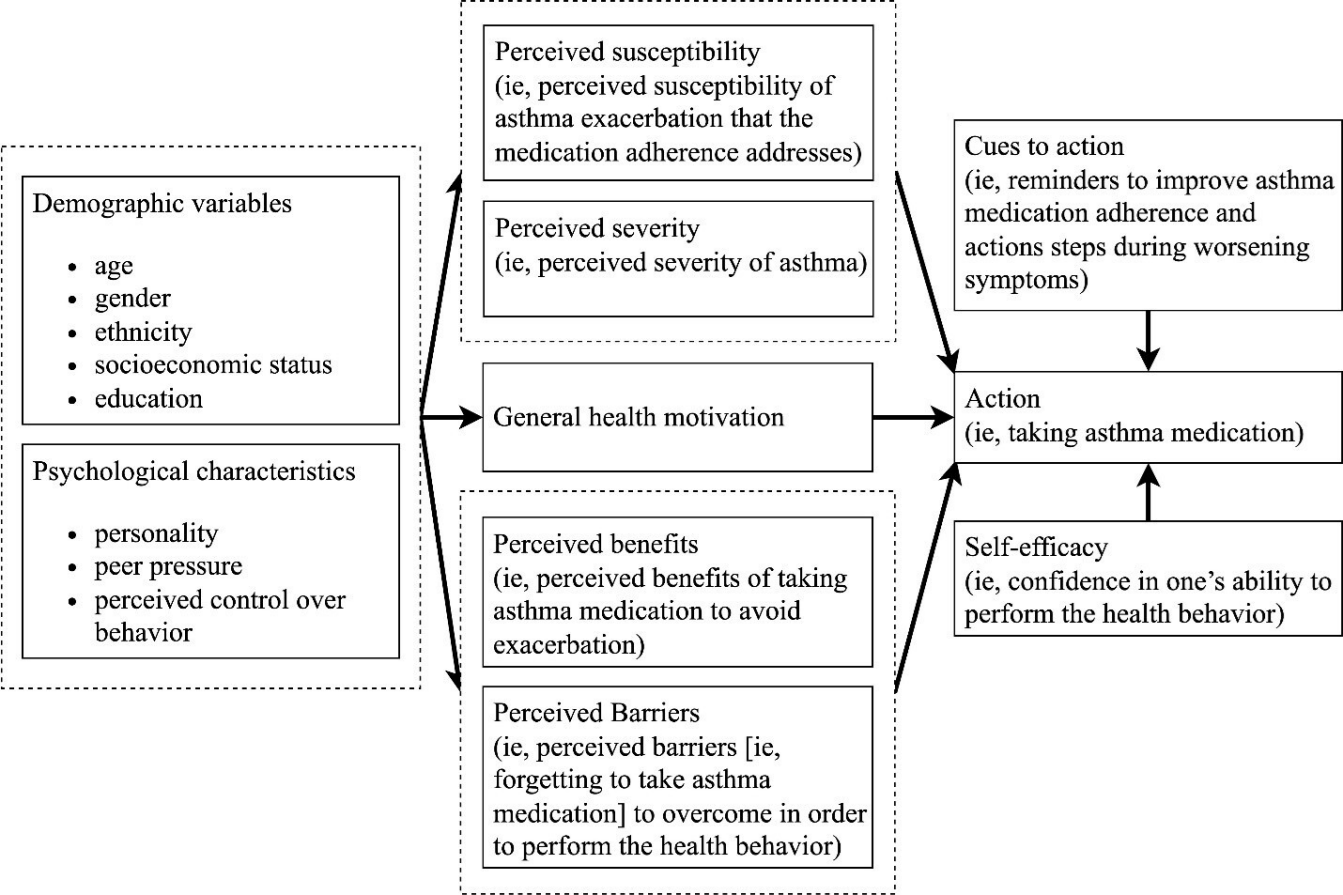
Design of Pulmonary Education and Knowledge Mobile Asthma Action Plan according to the health belief model.

We designed the functionalities of PEAK-mAAP based on the HBM to affect behavior change (ie, asthma self-management based on prescribed AAP). Figure 1 depicts how the design of PEAK-mAAP addresses the different constructs of the HBM. Notably, the HBM integrates considerations of individual demographics and psychological traits—termed modifying variables. These variables indirectly influence health-related actions by impacting factors like health motivation, perceived risks, severity, benefits, and obstacles.

After the preliminary design of PEAK-mAAP, we engaged in iterative enhancements by involving all stakeholders through a UCD approach. We convened a community advisory board with our intended end users, that is, adolescent (ie, teens and emerging adults aged between 12 and 20) patients with asthma, their caregivers, asthma care providers, UCD specialists, and researchers. community advisory board members provided feedback on the features of mAAP and iterative feedback and beta testing of the final app.

### Implementation of PEAK-mAAP

**Figure 2.**
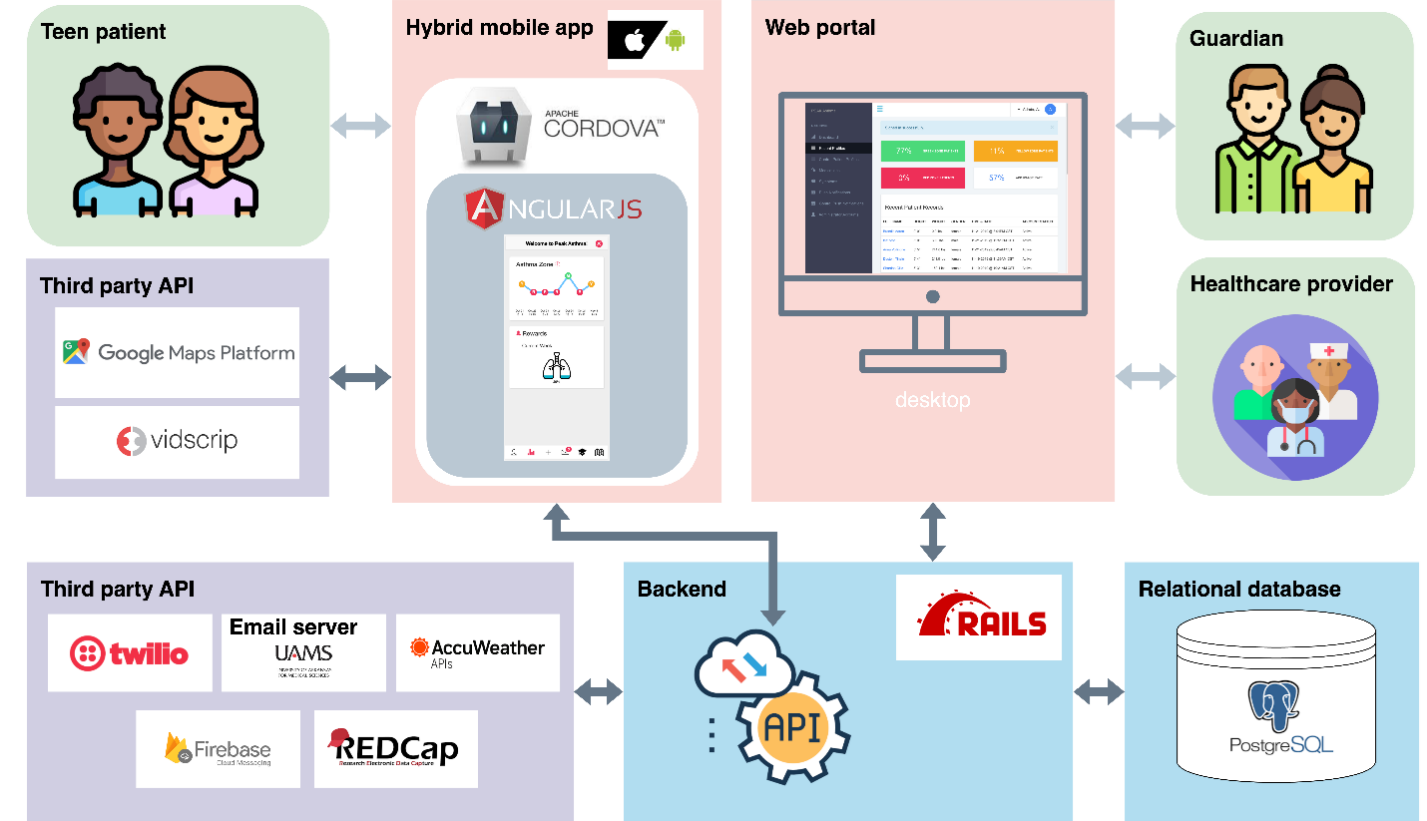
The 5-component system architecture which supports Pulmonary Education and Knowledge Mobile Asthma Action Plan and the web portal. API: application programming interface; REDCap: Research Electronic Data Capture.

PEAK-mAAP is a hybrid mobile app developed using standard web technologies and housed within a native container—specifically Apache Cordova—for adolescents. The web portal, designed for health care providers and guardians of adolescents, is built with Ruby on Rails [17]. Figure 2 illustrates the 5-component system architecture that supports the functionality of the app and the web portal.

First, a relational database, PostgreSQL [18], an advanced open-source object-relational database system, is employed to manage and categorize a range of data, including patient demographic details, guardian and provider profiles, medication records, and specific activity logs such as peak flow recordings. In addition, the database houses preset content, like educational tips and instructional videos. To safeguard against any potential data loss, regular backups are performed.

Second, our backend, built on the robust Ruby on Rails framework, offers RESTful application programming interfaces (APIs) to bridge our hybrid mobile app with its backend database. It also integrates with 5 external APIs, like weather, to enrich the mobile app’s features. Besides, adhering to web development best practices, this backend employs the model-view-controller design pattern to support the web portal. The web portal is tailored for adolescent health care providers such as doctors, nurses, and researchers, as well as guardians like parents and grandparents, enabling them to monitor PEAK-mAAP usage effectively.

Third, we implemented PEAK-mAAP as a hybrid mobile app to aid adolescents in managing their asthma. This approach involved the development of a cross-platform web app, encapsulated within a native app container. The hybrid mobile app was crafted using Apache Cordova and AngularJS (Google) [19] (ie, an open-source JavaScript framework primarily used for building dynamic single-page web apps). This allows programmers to use standard web technologies such as HTML5, CSS3, and JavaScript for cross-platform development.

Fourth, we engineered a model-view-controller–based web app to facilitate monitoring adolescents’ mobile app usage by both health care providers and guardians. Health care providers can access a comprehensive view of all patients’ information, mobile app usage, and asthma-related activities through a specialized web portal. Concurrently, guardians are provided a tailored view allowing them to observe their children’s information and interactions with the PEAK-mAAP, reinforcing transparency and personalized care.

Fifth, to enhance our mobile app for adolescents’ asthma self-management, we integrated 7 third-party APIs. Directly, we incorporated the Google Maps [20] API for locating hospitals and pharmacies and Vidscrip (Clear.md Inc) [21] for educational asthma videos. Indirectly, through our Ruby on Rails backend, we added the Twilio [22] API for alert calls and texts during severe asthma exacerbation; an email server hosted by the University of Arkansas for Medical Sciences to notify health care providers and guardians; the AccuWeather [23] API for real-time weather and pollen levels; the Firebase Cloud Messaging [24] API for cross-platform notifications; and the REDCap (Research Electronic Data Capture; Vanderbilt University) [25,26] API for automated monthly survey distributions. These integrations ensure a holistic approach to asthma management for adolescents.

### Usability Assessment

The PEAK-mAAP usability assessment was conducted over two months, spanning January and February 2019, before the formal enrollment of research participants in the subsequent RCT. Usability assessment is a widely applied method across various industries, including aviation, automotive, and software development, aimed at enhancing system functionality by assessing an app’s effectiveness and efficiency. Effectiveness refers to the extent to which users can successfully accomplish tasks and achieve their goals, while efficiency evaluates the time and effort required to complete a given task.

Participants were selected using a convenience sampling method and a total of 13 individuals, ranging from 12 to 20 years old in age, were recruited. All participants had a confirmed clinical diagnosis of asthma, as documented in their medical records. Recruitment efforts targeted potential candidates through 2 primary channels: the Arkansas Children’s Research Institute database, which provided access to a broad pool of pediatric patients with existing asthma diagnoses, and direct referrals from health care providers familiar with eligible patients. To qualify for the usability assessment, participants were required to meet specific eligibility criteria designed to ensure they could effectively engage with the PEAK-mAAP system. Inclusion criteria consisted of (1) a current diagnosis of asthma, verified by a health care professional; (2) previous exposure to or familiarity with AAPs, to facilitate comparative feedback on the mobile app; (3) proficiency in English to ensure clear communication during the assessment and accurate comprehension of the app’s content; and (4) the absence of any visual or auditory impairments that could hinder the ability to interact with the app’s interface. Individuals were excluded if they had significant respiratory conditions other than asthma, such as cystic fibrosis or chronic obstructive pulmonary disease, which could introduce variability in the management strategies assessed. In addition, participants with developmental delays or cognitive impairments that could interfere with their ability to comprehend instructions, navigate the app, or provide reliable feedback were not eligible for the study. The sample size of 13 was deemed sufficient, adhering to the standard usability principle that even 5 participants can identify the most severe usability issues and 80% of all usability issues [27].

To ensure consistency, all participants were provided with the same iPad (Apple Inc) device for testing and watched a prerecorded demonstration video of the PEAK-mAAP app. Following the video, participants were instructed to explore the app using the study iPad to familiarize themselves with its features. Subsequently, they were asked to complete eight tasks using the app: (1) logging Peak Flow, (2) logging Symptoms, (3) setting a reminder for logging their Peak Flow or symptoms, (4) reading daily tips and viewing educational videos, (5) reading notifications, (6) describing directions or locations of the hospital or pharmacy displayed on the map, (7) describing the weather for patient’s location, and (8) reporting number of days they logged and rewards within the app. A stopwatch was used to time each user while performing each task or scenario. Any extra steps performed during the scenarios were recorded to determine if there were common mistakes or additional steps taken for any of the app’s features. During this process, users were asked to adopt the think-aloud [28] process by verbalizing any trouble they had with the app, features they found exciting or beneficial, as well as features that were confusing or not useful. This approach provided evaluators with deeper insights beyond those obtainable through standard cognitive walkthroughs. There were at least 2 evaluators in the room during each usability test session. While observing the user, evaluators tracked issues and comments, and then aggregated the information from all usability tests. Observed errors were categorized using Nielsen-Shneiderman heuristics [29] (Table 1), a set of principles that provide guidelines and best practices for user interface design based on established usability heuristics.

During the study, evaluators assigned a severity rating to each identified issue, categorizing them into (1) cosmetic, (2) minor, (3) major, and (4) catastrophic. A cosmetic issue refers to a superficial concern that does not affect users’ ability to complete a task, such as formatting inconsistencies or color preferences; these issues are typically of lower priority for resolution. A minor issue represents a problem with an available workaround, such as restricting numerical input to whole numbers rather than allowing decimals. Major issues, however, can significantly impact data quality, user satisfaction, or system functionality. Examples include the inability to access a function by clicking an icon, necessitating the use of a menu bar instead, or encountering difficulty when attempting to exit a screen. These issues require prioritization. Finally, a catastrophic severity rating indicates a critical system failure that demands immediate remediation. For instance, a user’s inability to locate or input essential information constitutes a catastrophic issue.

**Table 1.** Nielsen-Shneiderman heuristics.

Error	Heuristic	Description
1	Consistency	Consistency and standards: Users should not have to wonder whether different words, situations, or actions mean the same thing. Standards and conventions in product design should be followed (*eg, font and layout and position*).
2	Visibility	Visibility of system state: Users should be informed about what is going on with the system through appropriate feedback and display of information (eg, what is current state of the system and what changes are made after an action).
3	Match	Match between system and the world: The image of the system perceived by users should match the model the users have about the system (eg, user model matches system image, and objects on the system should match objects of the task).
4	Minimalist	Any extraneous information is a distraction and a slow-down (eg, less is more and simple is efficient).
5	Memory	Minimize memory load: Users should not be required to memorize a lot of information to carry out tasks. Memory load reduces users’ capacity to carry out the main tasks (eg, recognition vs concrete examples such as DD/MM/YY, eg, 10/20/15).
6	Feedback	Informative feedback: Users should be given prompt and informative feedback about their actions (eg, response time, concrete and specific, and not abstract and general).
7	Flexibility	Flexibility and efficiency: Users always learn and users are always different. Give users the flexibility of creating customization and shortcuts to accelerate their performance (eg, shortcuts for experienced users, templates, and bookmarks).
8	Message	Good error messages: The messages should be informative enough such that users can understand the nature of errors, learn from errors, and recover from errors (eg, precise not vague or general and polite messages).
9	Error	Prevent errors: It is always better to design interfaces that prevent errors from happening in the first place (eg, avoid text wraps, avoid slips or mistakes, and interfaces that make errors impossible).
10	Closure	Clear closure: Every task has a beginning and an end. Users should be clearly notified about the completion of a task (eg, clear beginning, middle and end, and clear feedback that goal was achieved).
11	Undo	Reversible actions: Users should be allowed to recover from errors. Reversible actions also encourage exploratory learning (eg, reverse actions in a single action and prevent serious errors).
12	Language	Use users’ language: The language should be always presented in a form understandable by the intended users (eg, use standard meaning, language for specialized group, and users’ perspective).
13	Control	Users in control: Do not give users that impression that they are controlled by the systems (eg, users are initiators not responders to actions, and avoid surprising actions and tedious sequences).
14	Documentation	Help and documentation: Always provide help when needed.

### User Satisfaction Assessment

Satisfaction refers to the user’s contentment and attitude toward the system. After completion of the think-aloud process, users were asked to complete a System Usability Scale (SUS) [30] survey. The SUS is a short, 10-question survey tool with high reliability and validity that measures user satisfaction. The survey employs a Likert scale ranging from 1 (strongly disagree) to 4 (strongly agree) to generate a single composite score representing the app’s overall usability. Participants recorded their responses on paper, which were later digitized by the study team. At the end of the assessment, users were encouraged to share any concerns or recommendations.

### Ethical Considerations

This study was approved by the University of Arkansas for Medical Sciences Institutional Review Board (protocol #206110). Informed consent (Multimedia Appendix 1) was obtained from all participants after explaining the study’s purpose and procedures. Participant data were deidentified to ensure privacy and confidentiality. Each participant received US $50 as compensation for their time and involvement.

## Results

### Design of PEAK-mAAP

Using the HBM to explain current behaviors within our population, we hypothesize that the “cues to action” provided by PEAK-mAAP will be an essential factor in triggering behavior change. The app provides asthma educational video modules to address the “perceived susceptibility and severity” of asthma. The personalized mobile app features also provide additional “cues to action” during worsening symptoms to address “perceived benefits and barriers” (ie, give a clear action plan for the users to manage current asthma symptoms). Based on the education provided combined with self-management assistance from PEAK-mAAP, we hypothesize that self-efficacy and adherence will improve and result in improved asthma control for PEAK-mAAP users. To prove our hypotheses, we are conducting an RCT. The results of the RCT will be published upon completion of the trial.

### Final Implementation of PEAK-mAAP

PEAK-mAAP is structured around 7 key modules.

**Figure 3.**
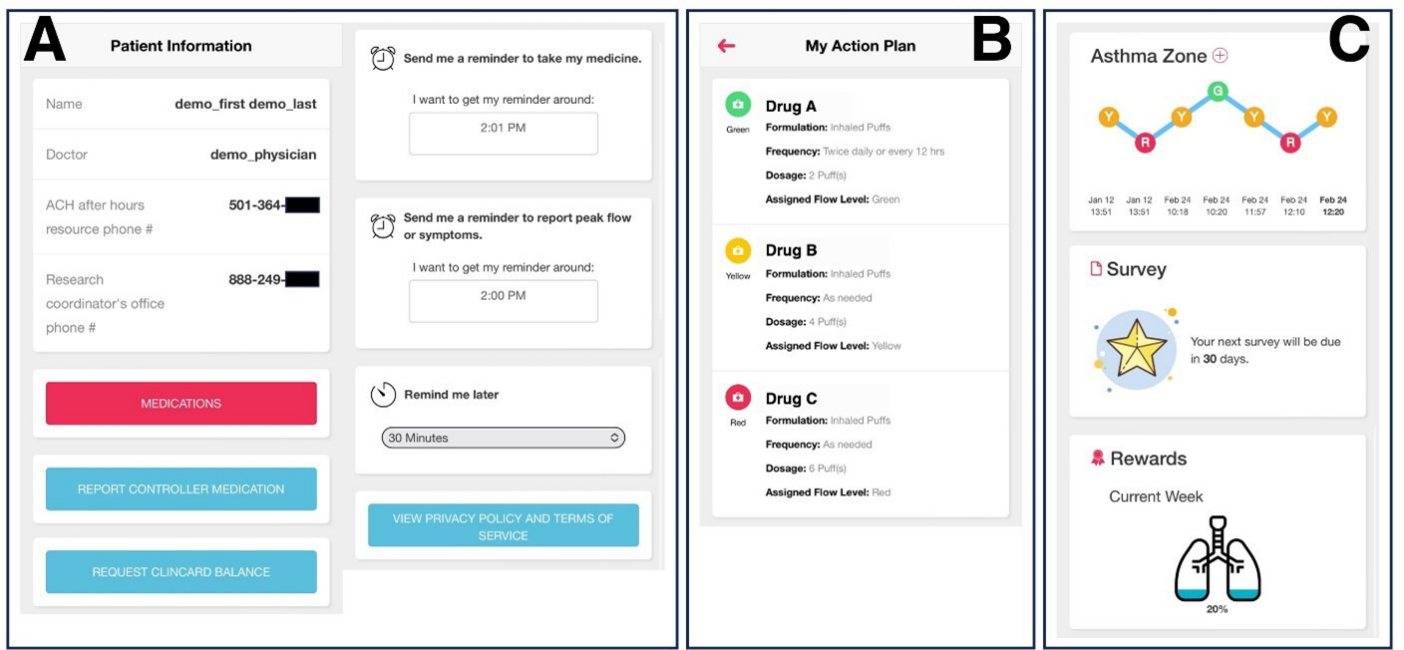
(**A**) The patient information tab; (**B**) the patient medication page with the patient’s personalized Asthma Action Plan; and (**C**) the asthma zone record line chart, the survey countdown module, and the reward progress tracker.

First, as illustrated in Figure 3A, the patient information module encompasses a table presenting patient and health care provider details; a red “MEDICATIONS” button linking to the patient’s asthma medication plan; a blue “REPORT CONTROLLER MEDICATION” button for logging medications; a “REQUEST CLINCARD BALANCE” button for users to request their ClinCard’s balance, a reloadable debit card for research participant incentive; customizable reminder settings; and a “VIEW PRIVACY POLICY AND TERMS OF SERVICE” button to access the PEAK-mAAP’s privacy terms. Figure 3B highlights a patient’s personalized AAP, guiding actions including controller medication and rescue medication use based on their asthma zone, whether green, yellow, or red.

Second, a dashboard of patient activities, as seen in Figure 3C; the “Asthma Zone” allows visualization of the patient’s recent asthma zones through a line chart, where each zone is depicted by uniquely colored circles. For color-blind users’ ease, each circle also carries a letter mark indicating the logged zone. This intuitive design lets patients swiftly gauge their recent asthma status. The “Survey” card indicates the days leading up to the RCT’s monthly survey due date. The “Rewards” card tracks weekly progress toward participant logging goals. As part of the PEAK-mAAP RCT trial, users earn nominal monetary incentives for active app engagement each week. The first daily record of a Peak Flow value or symptom will begin to fill the lung icon, signifying task completion and eligibility for the day’s incentive. Completion of tasks each day will increase filling the lungs until the week’s goals (eg, logging daily or completing the RCT’s monthly survey) are met and the lungs fill completely. Such tangible rewards can bolster users’ adherence to the app.

**Figure 4.**
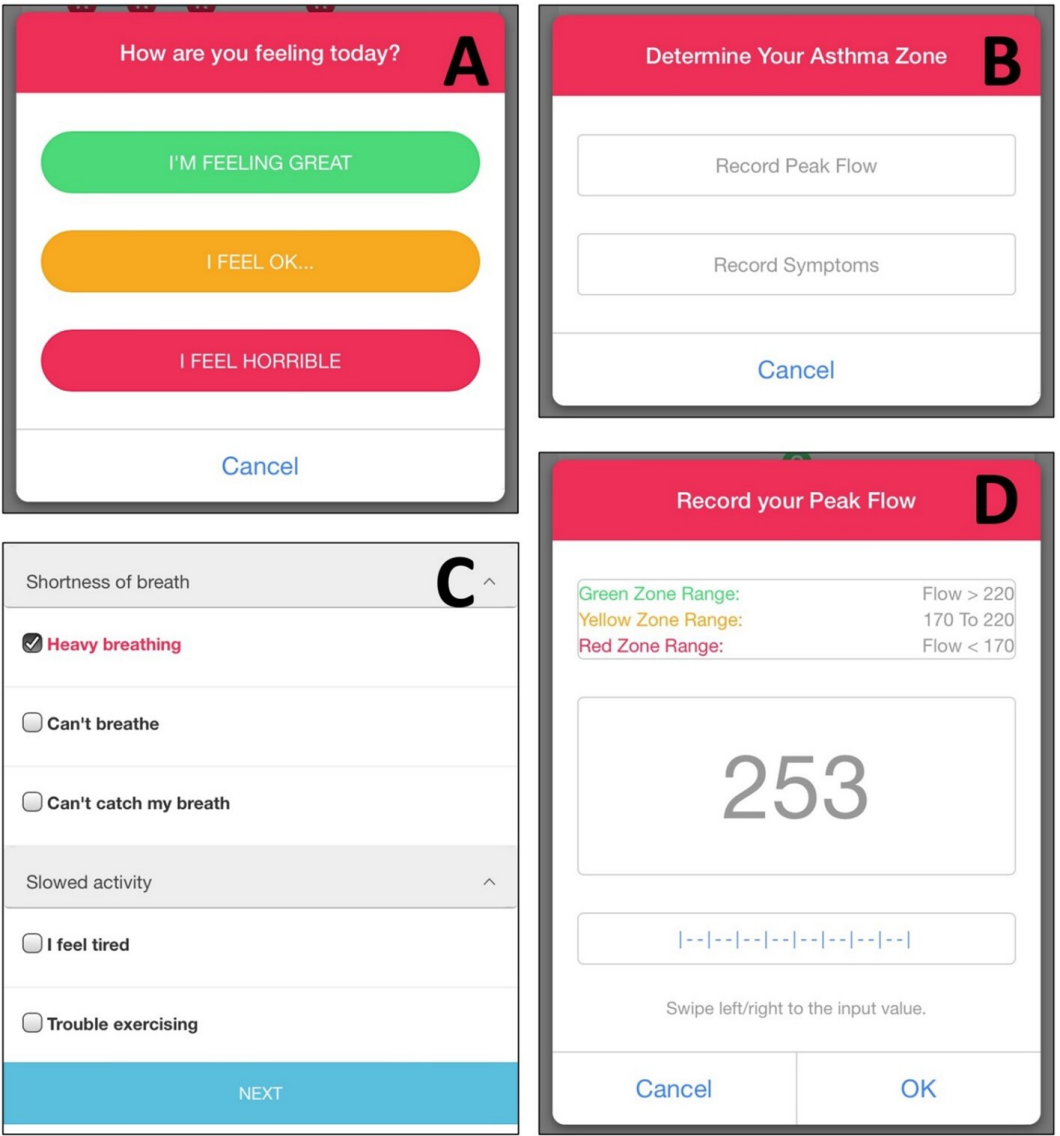
Pulmonary Education and Knowledge Mobile Asthma Action Plan navigates the patient through a series of simple steps to log daily asthma status and during any episodes of increased symptoms to determine their asthma zone: (**A**) “How are you feeling today?”; (**B**) “Determine your Asthma Zone”; (**C**) “Record your Peak Flow”; and (**D**) symptoms recording page. Patients can elect to enter either Peak Flow or symptoms at their preference.

Third, a Peak Flow or asthma symptom record module: based on self-recorded Peak Flow value or asthma symptoms, PEAK-mAAP will classify the patient into their personalized asthma zones (ie, green, yellow, and red zones) and give zone-specific instructions (ie, action plan) such as taking emergency medications for acute symptoms. As shown in Figure 4A, before recording asthma-related symptoms or Peak Flow value, the system will first ask the participant “How are you feeling today?” The options are (1) “I’m feeling Great,” (2) “I feel OK,” and (3) “I feel horrible.” Afterward, as shown in Figure 4B, the patient is asked to select how they prefer to determine their asthma zone, either Peak Flow or asthma symptoms. Figure 4C depicts the “Record your Peak Flow” screen, in which the patient can swipe on the blue bar to change the Peak Flow value. When the patient wants to report symptoms, a screen shown in Figure 4D will pop up, and the patient selects from a panel of 6 groups of symptoms to report.

**Figure 5.**
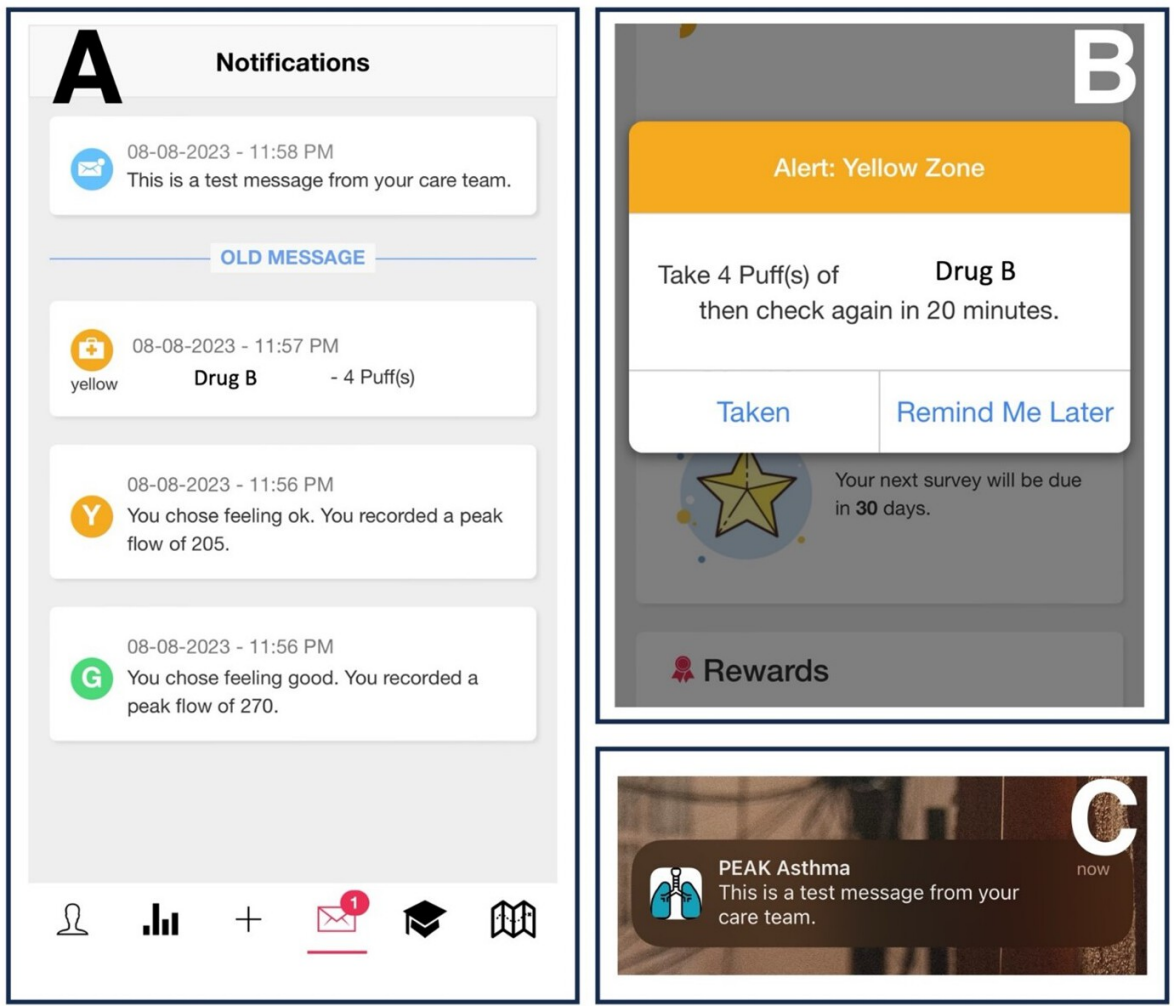
(**A**) The history of notifications that were sent to the patient; (**B**) the in-app notification screen; and (**C**) the mobile system’s native notifications*.*

Fourth, a notification module, as seen in Figure 5A, which shows a list of example notifications. The most recent messages are on the top of the list, and the unread and read notifications are separated by an “OLD MESSAGE” line separator. Figure 5B is an example of the in-app notification and Figure 5C is an example of the push notification to mobile phone native notification system.

**Figure 6.**
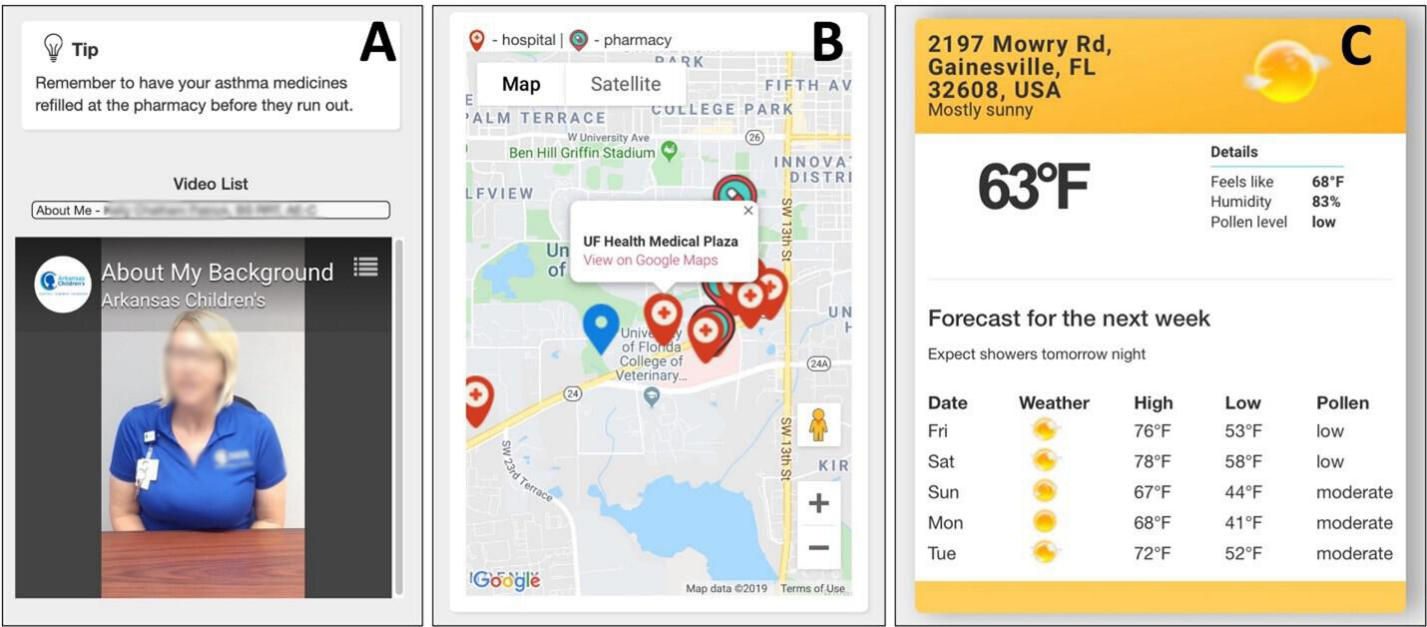
(**A**) The education module; (**B**) the nearby medical help map; and (**C**) the weather information module.

Fifth, an education module, as shown in Figure 6A, which consists of (1) an asthma educational tip card and (2) a list of asthma educational videos. The educational tip card will be refreshed weekly and the tips will also be pushed to the patients through the notification function. The asthma educational videos were created by the research team including asthma clinicians, asthma educators, and respiratory therapists using the Vidscrip platform. These resources aim to heighten patient awareness and empower them to manage their asthma more effectively.

Sixth, a nearby medical help map, as seen in Figure 6B, displaying a Google Map pinpointing nearby medical facilities, including hospitals and pharmacies. Users can click on markers for more detailed information about a facility and also receive directions via the Google Maps mobile app.

Seventh, a weather information module, as shown in Figure 6C, consisting of a weather information card, which shows real-time weather data and pollen level information that may affect patients’ asthma outcomes.

### Web Portal

We developed a web portal for the health care providers and guardians of the patients to enable monitoring of the patient’s usage of PEAK-mAAP and keep track of patients’ asthma status. The health care providers are granted full access, which means they can view all the patients’ information and see the statistics related to the use of PEAK-mAAP. Furthermore, health care providers can create patient accounts, edit the information of the patients, and send notifications to all patients or a specific one. On the other hand, guardians are only allowed to access limited information specifically related to the patient they are associated with.

### Effectiveness and Efficiency of PEAK-mAAP

During the think-aloud process, evaluators recorded a total of 39 instances in which participants encountered difficulties in correctly performing a task, referred to as errors. These errors corresponded to violations of 9 out of the 14 heuristics under evaluation. The most frequently violated heuristic principles were match (n=12) and visibility (n=8). These were followed by memory (n=5), control (n=4), and language (n=3). Additional violations were observed in the closure, minimalist, document, and error heuristics. Examples of errors related to the match principle included confusion between the notification tab and the reminder tab, as well as difficulties in navigating the map to locate hospitals and pharmacies. The visibility principle was the second most frequently violated, with errors such as difficulty distinguishing between old and new messages within the app and challenges in locating and logging symptoms that could trigger relevant notifications. A memory error example was that a participant could not remember how to log their activity for the week to receive a reward. An example of a control error was that the participant had trouble recording their peak flow using the dial to select the correct number. Not understanding the icons used on the map was an example of a language error. There were 5 principles that were not violated, that are, undo, message, consistency, feedback, and flexibility. Of the 39 total errors, 26% (n=10) were rated as minor, 69% (n=27) as major, and 5% (n=2) as catastrophic.

### Satisfaction Assessment of PEAK-mAAP

Participant satisfaction with the PEAK-mAAP mobile app was assessed using the SUS, yielding an overall mean score of 83 (SD 5.54), indicating high usability satisfaction. Older participants (≥17 years old) reported higher usability scores, with a mean of 87.5 (SD 1.71), compared with younger participants (12‐16 years old), who had a mean score of 77.5 (SD 2.36). These findings suggest that older users found the app more user-friendly. The highest agreeability was found in survey question 7 stating, “*I would imagine that most people would learn to use the app very quickly.*” In total, 12 participants reported that they strongly agreed with this statement, and the remaining responses indicated that they agreed with this statement. The greatest variability was seen in survey question 10 stating, “*I needed to learn a lot of things before I could get going with the app*” (strongly disagree=6, disagree=2, neither or neutral=2, agree=1, strongly agree=2).

## Discussion

### Principal Findings

The primary objective of this study was to develop and evaluate PEAK-mAAP, an innovative mHealth app designed to improve asthma self-management among adolescents. Findings indicate that PEAK-mAAP, rooted in the HBM and developed using a UCD approach, enhances adolescents’ engagement in asthma management. The usability assessment provided positive feedback on the app’s ease of use and its potential for supporting behavior change. These results align with our initial hypotheses that a well-integrated mHealth platform—combining educational content, symptom tracking, and behavior modification strategies—can effectively promote self-management practices.

### UCD and Usability Assessment

A key factor in PEAK-mAAP’s potential success is its UCD approach, which ensures the app is intuitive and user-friendly. By incorporating user feedback early in development, the app was designed to meet the needs and expectations of adolescents, thereby encouraging consistent use. When users feel that an app is tailored to their needs, they are more likely to engage with it over time. The usability assessment results reinforced this; participants rated the app highly in usability, achieving an impressive usability score. Overall user satisfaction with PEAK-mAAP was high. The SUS scores averaged 83 (SD 5.54), with older adolescents (>17 years) rating it higher (87.5, SD 1.71) than younger participants (77.5, SD 2.36). This trend may indicate that older adolescents are generally more comfortable using mobile apps, or that certain app features are more intuitive for them. Regardless of age, participants agreed that most users would be able to learn how to use the app quickly.

PEAK-mAAP performed well in many heuristic categories. Users encountered no issues with consistency, feedback, flexibility, message, or undo. However, errors in “match” (12/39, 31%) and “visibility” (8/39, 21%) posed significant challenges. The “match” errors stemmed from inconsistencies between user expectations and actual system functions, particularly regarding reminders and navigation tools. Meanwhile, “visibility” issues were especially problematic, as some users struggled to distinguish new messages from old ones. Notably, 2 visibility errors were classified as catastrophic, as they significantly impacted user interaction with notifications.

However, despite strong usability performance, testing identified critical issues that could impact the user experience if left unaddressed. For example, users had difficulty identifying the correct icon to set Peak Flow logging reminders—many mistakenly selected the notification icon instead. In addition, the dial interface for recording Peak Flow was not user-friendly, making it challenging for users to input accurate data. This issue was particularly significant because the peak flow value determines the personalized asthma management guidance provided by the app. Younger participants also faced challenges with reading the map, likely due to their limited experience with navigation tools.

### Identified Usability Issues and Modifications

To enhance usability and ensure a seamless experience, several modifications were made before the RCT. The reminder icon was redesigned to align with users’ expectations, and the notification icon was changed from a bell to an envelope to prevent confusion. Similarly, the dial’s sensitivity was adjusted to allow for more precise Peak Flow input, reducing the risk of inaccurate data entries. To address visibility concerns, new messages on the notification screen were color-coded (blue for unread messages, gray for read ones), and a dividing line was added to separate them. In addition, on the login screen, a password reset option was implemented to improve accessibility. Another significant modification was made to the educational content delivery. Initially, weekly asthma education videos under the education tab played consecutively, which could be overwhelming. This feature was adjusted to allow users to control playback, preventing automatic continuous play. For motivation and engagement, the app incorporated an age-appropriate reward system, represented by a progressively filling blue lung icon that visually tracked the user’s daily logging progress. Finally, usability feedback also informed the development of training materials to better introduce participants to the app’s features, ensuring that users fully understood its capabilities.

### Advantages of the Hybrid Mobile App Approach

The decision to develop PEAK-mAAP as a hybrid mobile app brings several benefits. Hybrid apps work across different platforms, meaning more people can use the app without compatibility issues. This approach is also cost-effective since developers do not need to create separate versions for different platforms. Plus, hybrid apps can easily integrate with a phone’s built-in features, enhancing the user experience. Compared with a native implementation, where the app must be developed separately for Android and iOS platforms, the hybrid approach significantly shortened the development time frame. It facilitated a rapid reach to a broader user base and proved to be more cost-effective in construction and maintenance. Furthermore, the hybrid mobile app can run on browsers, ensuring that adolescents remain engaged with PEAK-mAAP even if access to the mobile platform is unavailable, such as in the case of a lost phone.

### Limitations

Despite these promising outcomes, several limitations must be acknowledged. Some users experienced an initial learning curve, which could impact short-term engagement. Longitudinal studies are needed to assess sustained usage and long-term effectiveness. Furthermore, as the efficacy of PEAK-mAAP has not yet been tested through an RCT, conclusions regarding its impact on clinical outcomes remain speculative at this stage.

### Conclusion

This study presents the design, development, and usability evaluation of PEAK-mAAP, a hybrid mHealth intervention designed to support asthma self-management in adolescents. Rooted in the HBM and developed through a UCD approach, PEAK-mAAP integrates educational content, symptom tracking, medication adherence tools, and real-time feedback to enhance self-management behaviors. Usability testing revealed high user satisfaction, with strong SUS scores and positive engagement. While some challenges—particularly in navigation, reminders, and visibility—were identified, subsequent modifications improved the user experience before launching the RCT. The hybrid mobile app approach ensures cross-platform compatibility, cost efficiency, and accessibility, positioning PEAK-mAAP as a scalable and adaptable solution for adolescent asthma management. Although usability results are promising, further research should assess long-term effectiveness and sustainability in improving asthma control through a rigorous RCT. Addressing usability concerns and incorporating ongoing user feedback will be critical to fostering widespread adoption and adherence among adolescents. Ultimately, PEAK-mAAP represents a significant advancement in mHealth technology, bridging gaps in adolescent asthma care with a personalized and evidence-based approach to self-management.

## Supplementary material

10.2196/64212 Multimedia Appendix 1 Usability testing agreement.

## Abbreviations

AAP: Asthma Action Plan
API: application programming interface
HBM: health belief model
mAAP: mobile health Asthma Action Plan
mHealth: mobile health
PEAK-mAAP: Pulmonary Education and Knowledge Mobile Asthma Action Plan
RCT: randomized controlled trial
REDCap: Research Electronic Data Capture
SUS: System Usability Scale
UCD: user-centered design
