# Supplementary material for: Mobile Health App for Adolescent Asthma Self-Management: Development and Usability Study of the Pulmonary Education and Knowledge Mobile Asthma Action Plan
Source: JMIR Form Res. 2025 Apr 24;9:e64212. doi: 10.2196/64212 (PMC12045068; doi:10.2196/64212)
Supplement: Multimedia Appendix 1 [file formative-v9-e64212-s001.pdf]

Usability Test Agreement

Thank you for agreeing to participate in our usability test for the new PEAKmAAP asthma NutriMap mobile applications.

Dr. Tamara Perry and her research team will be taking notes and recording today's session to allow our research team to observe and benefit from any feedback you provide during the usability test.

Please sign below to agree to be a participant in this usability test.

I, ___________________________________Acknowledge that I am volunteering to participate in a usability test for a mobile asthma application.

___________________________	___________________
Participants Signature	Date
___________________________	___________________
Parent/Caregiver Signature	Date
___________________________	___________________
Research Coordinator	Date
